# Systematic Review of *Wolbachia* Symbiont Detection in Mosquitoes: An Entangled Topic about Methodological Power and True Symbiosis

**DOI:** 10.3390/pathogens10010039

**Published:** 2021-01-06

**Authors:** Luísa Maria Inácio da Silva, Filipe Zimmer Dezordi, Marcelo Henrique Santos Paiva, Gabriel Luz Wallau

**Affiliations:** 1 Departamento de Entomologia, Instituto Aggeu Magalhães (IAM), Fundação Oswaldo Cruz (FIOCRUZ), Av. Professor Moraes Rego, s/n, Campus da UFPE, Cidade Universitária, Recife 50740-465, Brazil; zamaria1996@gmail.com (L.M.I.d.S.); zimmer.filipe@gmail.com (F.Z.D.); 2 Núcleo de Bioinformática (NBI), Instituto Aggeu Magalhães (IAM), Fundação Oswaldo Cruz (FIOCRUZ), Recife 50670-420, Brazil; 3 Núcleo de Ciências da Vida, Universidade Federal de Pernambuco (UFPE), Centro Acadêmico do Agreste-Rodovia BR-104, km 59-Nova Caruaru, Caruaru 55002-970, Brazil

**Keywords:** *Wolbachia* detection, mosquito, symbiosis, methods, genotyping

## Abstract

*Wolbachia* is an endosymbiotic bacterium that naturally infects several arthropods and nematode species. *Wolbachia* gained particular attention due to its impact on their host fitness and the capacity of specific *Wolbachia* strains in reducing pathogen vector and agricultural pest populations and pathogens transmission. Despite the success of mosquito/pathogen control programs using *Wolbachia*-infected mosquito release, little is known about the abundance and distribution of *Wolbachia* in most mosquito species, a crucial knowledge for planning and deployment of mosquito control programs and that can further improve our basic biology understanding of *Wolbachia* and host relationships. In this systematic review, *Wolbachia* was detected in only 30% of the mosquito species investigated. Fourteen percent of the species were considered positive by some studies and negative by others in different geographical regions, suggesting a variable infection rate and/or limitations of the *Wolbachia* detection methods employed. Eighty-three percent of the studies screened *Wolbachia* with only one technique. Our findings highlight that the assessment of *Wolbachia* using a single approach limited the inference of true *Wolbachia* infection in most of the studied species and that researchers should carefully choose complementary methodologies and consider different *Wolbachia*-mosquito population dynamics that may be a source of bias to ascertain the correct infectious status of the host species.

## 1. Introduction

*Wolbachia pipientis* is an endosymbiotic bacteria from the *Rickettsiales* order, identified for the first time in 1923 in *Culex pipiens* ovaries [1]. Since then, *Wolbachia* has been widely studied from basic to applied biology. It is estimated that strains of the genus *Wolbachia* are naturally present in 66% of all insect species, showing a wide array of ecological interactions, varying from parasitism, commensalism and mutualism, with their eukaryotic host cells [2,3,4,5]. In past years, several studies have been published focusing on *Wolbachia’s* ability to manipulate their host reproductive system due to the applicability of different derived phenotypes in new strategies to control arthropod species populations [6,7]. Different *Wolbachia* strains can generate parthenogenesis, feminization and cytoplasmic incompatibility (CI) on their hosts [8,9]. Such phenotypes increase the frequency of host infected individuals consequently enhancing the *Wolbachia* transmission to their progeny [10]. Moreover, *Wolbachia* can also be transmitted and infect new host species through horizontal transfer, that is, the transfer of *Wolbachia* to new individuals/species through other means than sexual intercourse. Such phenomenon is also known as host swift [11,12]. Therefore, the large host taxa breadth that *Wolbachia* currently infects is a result of successful lineages that are able to exploit vertical transmission and/or horizontal transfer inheritance modes [13,14].

*W. pipientis* is considered the only species of the genus *Wolbachia*, but major supergroups and lineages were proposed to classify the large genomic diversity of the different strains characterized so far [15]. Seventeen different *Wolbachia* supergroups ranging from A to R (except G) have so far been defined based on genome differences—mainly 16 s ribosomal region phylogenetic analysis [16,17,18,19]. The supergroups A and B are the most common supergroups found in arthropods, while the supergroups C and D are usually found in filarial nematodes [20,21]. Besides the classification into supergroups/lineages, *Wolbachia* can also be classified into strains differentiated based on genomic divergence and different effects they cause in their hosts.

The ability of different *Wolbachia* strains to modify the physiology of their hosts has been extensively explored as a biotechnological tool for insect population control [22,23]. Currently, biological control using *Wolbachia* is based on the management of two phenotypes that emerged from the crossing of *Wolbachia*-infected strains with natural mosquito populations: the first occurs when males infected with the bacteria are released into the environment to reproduce with *Wolbachia*-free females, which leads to CI between gametes, and the absence of viable offspring [6,24,25]. Continued release of infected males over time reduces the target mosquito population in a given site. This strategy is commonly known as the incompatible insect technique (IIT) [26,27,28,29]; in the second approach, the replacement strategy, females infected with a specific strain of *Wolbachia* that can reduce the replication of arthropod-borne viruses (arboviruses) such as Dengue (DENV), Zika (ZIKV), Chikungunya (CHIKV) and Yellow fever (YFV) [30,31,32,33,34,35] are released in the environment to replace the local natural mosquito population [6]. IIT has been deployed effectively in some cities in the United States focusing on *Ae. albopictus* and *Ae. aegypti* population control [36,37,38], in Tahiti with *Ae. polynesiensis*, in Singapore with *Ae. aegypti* and with *Ae. albopictus* in China in combination with the sterile insect technique (SIT) [28,39,40]. Both IIT and the replacement strategy relies on a key premise: that natural populations of the target species are *Wolbachia* free and/or not infected with the strain being released [41]. *Wolbachia* strains naturally infecting the mosquito target species might render both *Wolbachia* control strategies ineffective, therefore precise information about *Wolbachia* infection in mosquitoes is crucial for any planned deployment of such strategies [41].

Given that these strategies to control mosquito populations or pathogen transmission rely on curated information about the *Wolbachia* status of mosquito species and natural populations, prior knowledge and continued monitoring of the presence of *Wolbachia* in mosquitoes is crucial to plan and implement such control programs. *Wolbachia* can be detected through various molecular techniques that show variable sensitivity and specificity, such as polymerase chain reaction (PCR) (with specific and/or degenerate primers), PCR with various markers such as multilocus sequencing Typing (MLST), quantitative PCR (qPCR), microscopy methods, such as Fluorescent In Situ Hybridization (FISH), electron microscopy, and others [42,43,44,45]. The popularization of molecular methods has broadened the capacity of many laboratories to perform *Wolbachia* DNA detection in several insect species while the different microscopy techniques have been used by a small number of researchers [46]. Each of these techniques has particular advantages and limitations that can influence the detection of different *Wolbachia*-derived molecules and/or a true *Wolbachia* infection. Moreover, several well-known biological phenomena emerged from a symbiotic relationship must be considered when investigating *Wolbachia* infection: I—the variable infection rate of host species, that is, in most host species studied so far, *Wolbachia* strains infect only a fraction of the host population; II—multiple *Wolbachia* strains co-infection in the same individual; III—horizontal gene transfer of *Wolbachia* to the host genome and; IV—the large diversity of *Wolbachia* strains that might not be captured by all molecular techniques available [9,47,48].

This systematic review evaluated the presence of *Wolbachia* in culicids, analyzed the methods employed to detect the bacterium and provide guidelines and perspectives for researchers in this area.

## 2. Results

### 2.1. Articles

The 59 selected articles were published between the years of 2000 and 2020, with 2018 being the year with the highest number of publications (12). The average number of mosquito species evaluated for each article was 9.06, with 16.22 as the standard deviation. The largest number of species analyzed by a single article was 87, however, 37% of the studies evaluated only one species [49].

### 2.2. Methods Used to Detect Wolbachia in Culicids

*Wolbachia* has been screened using different methodologies that can be subdivided into two larger groups based on the molecule/cellular structure investigated: Amplification-based strategies: PCR, real-time PCR (qPCR), restriction fragment length polymorphism (RFLP), multilocus sequence typing (MLST), metabarcoding and Loop Mediated Isothermal Amplification (LAMP); and cell/structure visualization strategies: electron transmission microscopy (MET) and cell culture. Amplicon-based strategies have used several different target genes for *Wolbachia* detection, such as *wsp*, *fstz*, *16s rRNA*, *orf7* (from an integrated bacteriophage WO found in the *Wolbachia* genome), *Tr1*, *pk1*, *ank2*, *groE*, *18s rRNA*, *GP15*, *ISWpi1* transposable element, besides specific targets for the different strains of *Wolbachia*. While cell/structure visualization strategies relied on specific *Wolbachia* protein staining and/or the staining of the entire *Wolbachia* cell.

Of the total articles selected for this systematic review, 83% of them employed only one technique to detect *Wolbachia* in mosquitoes (Supplementary Material). The conventional PCR technique for more than one *Wolbachia* target gene was used in 51% of those with 4.16 targets per article on average, while 17 target genes was the highest number of target genes that was used in only a single article [49]. Of the articles that used only conventional PCR for amplification (20) of a single *Wolbachia* target gene, most of them chose the *wsp* gene, in 45% of the studies. Twelve studies used more than one PCR target genes as complementary methodologies and only two articles (16%) used more than one technique (Amplicon-based and Cell/Structure visualization) to detect *Wolbachia*. *Wolbachia* cell culture, metabarcoding, LAMP, and MET, were employed in only one article each [45,50,51,52] (Supplementary Material). It is important to note such highly heterogeneous and non-standardized use of different molecular biology techniques to detect *Wolbachia* in mosquitoes. Moreover, many of the methodologies employed alone are not able to differentiate between a true *Wolbachia* infection and the detection of *Wolbachia* molecule traces irrespective of the infection status (see Discussion section). Therefore, from now on we described the results collected in this review using a general term “*Wolbachia* detection” and the results should be taken cautiously regarding the *Wolbachia* infection status unless we stated that multiple methodologies have been employed corroborating a true *Wolbachia* infection.

### 2.3. Distribution of Wolbachia in Culicidae Species

**Figure 1:**
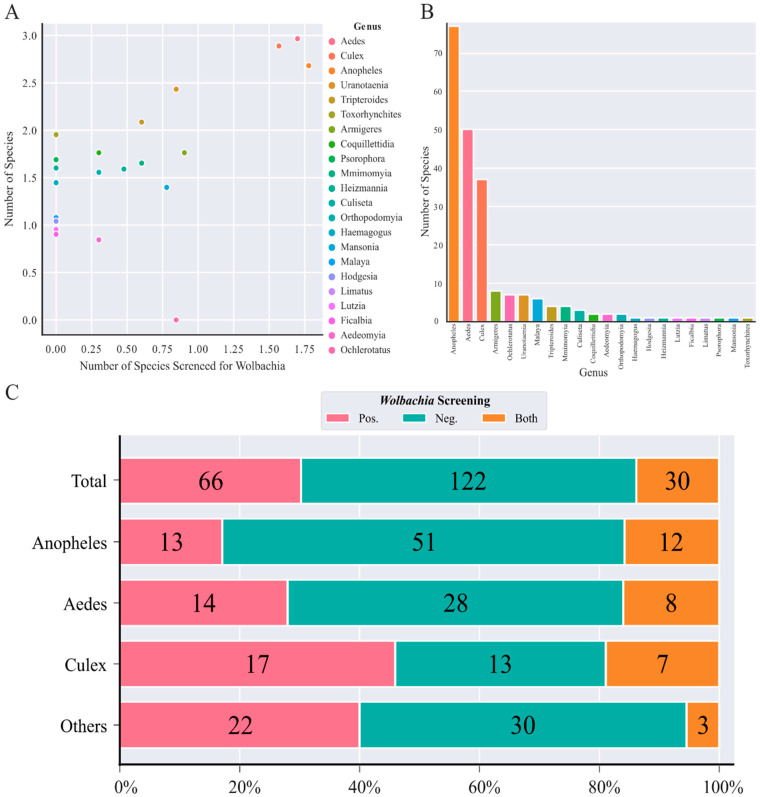
General information about *Wolbachia* screening on mosquitoes. (**A**) Correlation between the number of mosquito species studied for the presence of *Wolbachia* per genera and the number of mosquitoes species cotalogued per genera. Axis values are in log scale, Pearson’s correlation: 0.88. (**B**) Number of species per culicid genera screened by the presence of *Wolbachia*. (**C**): Distribution of 217 species by genera identified as positive and negative for *Wolbachia*. The *Anopheles*, *Aedes* and *Culex* genera were the most studied ones, the other genera were grouped in the “Others” category. The sum of separated values does not totalize 217 species, since *Ur.* Spp. is considered positive by one study and negative by other, but cannot be classified in “Both” categories, once we cannot guarantee if it corresponds to the same species.

Two hundred and seventeen Culicidae species belonging to 22 different genera were screened for *Wolbachia* so far, which corresponds to only 6% of all mosquitoes recorded [53]. *Anopheles*, *Aedes*, and *Culex* have the largest number of screened species, which correspond to 75% of all recovered species, with species of the *Anopheles* genus being the most abundant (76 species) (Figure 1B,C). Some genera such as *Ficalbia*, *Malaya*, *Haemagogus*, *Hodgesia*, and *Limatus* were represented by only one species but the species investigated from the last three genera were classified taxonomically only at genus level. *Ae. albopictus* and *Ae. aegypti* species were screened by *Wolbachia* in several studies, 26 and 21 different articles, respectively. There is a strong linear correlation between the number of mosquito species per genera and the number of species per genera investigated for *Wolbachia* infection (R = 0.883) (Figure 1A). An interactive map with all species screened for *Wolbachia* detection so far is available on the microreact platform (https://microreact.org/project/rxDRQdWjzg86eXCTF8oNn4).

Seventeen out of 22 genera had at least one species positive for *Wolbachia*, whereas the remaining five (*Culiseta*, *Haemagogus*, *Lutzia*, *Heizmannia* and *Mimomyia*) were negative. All species investigated so far from the *Coquillettidia*, *Limatus*, and *Psorophora* genera were *Wolbachia*-positive (Figure 1C).

Of the total species screened for *Wolbachia*, 122 were considered negative, and 66 were positive, which corresponds to approximately 56% and 30% of the total, respectively (Figure 1C). In addition, a different group of species (30 species—14%) were both considered positive and negative by different studies (Figure 1C, Supplementary Material). However, due to the high variability of the methods used in these studies (mainly amplicon-based), the infection status of most species needs further assessment.

### 2.4. Wolbachia Diversity and Infection Rate

**Figure 2:**
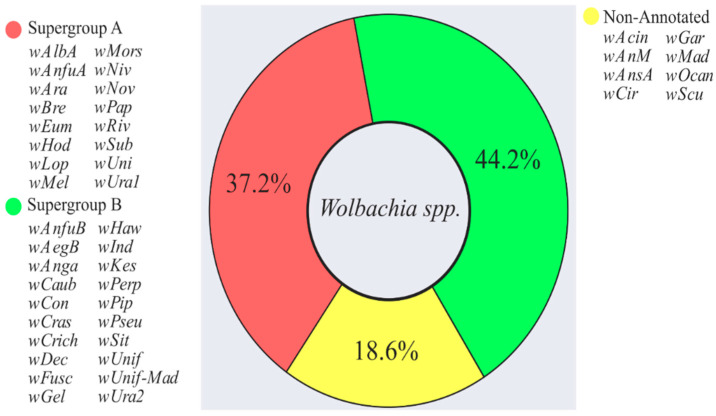
*Wolbachia* strains and its respective supergroups present on mosquitoes identified by this review.

Forty-four distinct strains of *Wolbachia* were characterized; 36% of those belong to supergroup A and 45% belong to supergroup B. The remaining 19% were not genotyped at the supergroup level (Figure 2). The most frequent strain found was *wPip* detected in 16 different mosquito species, followed by the *wCon* strain, detected in 9 species. In addition, some studies recorded the presence of *Wolbachia* from other supergroups (in addition to A and B), such as C in *Ae. aegypti*, D in *An. baimai*, and F in *An. minimus* and *An. maculatus*.

The infection rate, the percentage of *Wolbachia*-positive mosquitoes, was calculated for 32 out of 65 species mostly using amplification-based strategies (Supplementary Material). A large variation was found such as *Ae. albopictus* and *Ma. uniformis*, which showed infection rates varying from 15% to 100% (Table 1).

### 2.5. Infection Rate Variability between Studies Considering Widely Distributed and Studied Species

We observed that the detection of *Wolbachia* in culicids was carried out in mosquitoes from 52 countries, in Africa, Oceania, Europe, Asia, and the South, Central, and North America.

**Figure 3:**
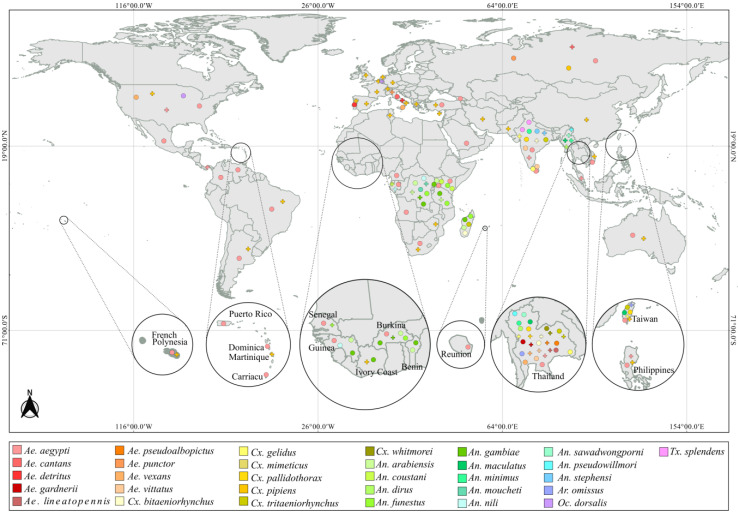
World map showing the species sampling site per country that had variable *Wolbachia* infection rate. Crosses and circles represent sites where the species were positive and negative, respectively. The full interactive map can be accessed at https://microreact.org/project/rxDRQdWjzg86eXCTF8oNn4.

Thirty-one species were considered positive by some studies and negative by others regarding the presence of *Wolbachia* (Figure 3). Twenty-one studies analyzed a total of 35 *Ae. aegypti* populations for the presence of *Wolbachia*, 31 populations were *Wolbachia* free. While four populations sampled in the USA, Malaysia, Thailand, India, Panama, and the Philippines were positive for *Wolbachia* from supergroups A, B, C, and *wAlbB* and *wAegB* strains. A similar pattern was seen for *An. gambiae*, evaluated in eight countries, in which populations from Burkina Faso and the Democratic Republic of Congo were *Wolbachia* free while two other studies reported infection, with the presence of *wAnga* strain in one of them. The same scenario occurred with *Cx. pipiens*, in which only one article registered the absence of *Wolbachia* in this species from Russia. The *Wolbachia* infection rate for *Ae. aegypti* and *An. gambiae* varied by site between 4.3% to 58% and 8% to 24%, respectively. It is possible that *Wolbachia* infection is limited to certain geographical regions and populations, but it is important to take into consideration the methodological approach employed by each study in order to confidently access the infection status of species and populations.

## 3. Discussion

Detailed knowledge of *Wolbachia* diversity and their ability to manipulate the host’s reproductive system and affect the replication of viruses have established *Wolbachia*-based vector and arbovirus control as one of the most promising strategies to mitigate the impact of mosquito-transmitted pathogens. Currently, there are two main approaches that use *Wolbachia* with this aim [37,102]: I—the replacement strategy that releases males and females of *Ae. aegypti* transfected with *Wolbachia* to replace the natural population to one that is refractory to several arboviruses [23]; II—the second approach is conducted by releasing male mosquitoes infected with *Wolbachia* seeking to induce CI and reduce the mosquito population [38]. To effectively deploy either strategy, researchers require precise information about any potential target mosquito species infected naturally by *Wolbachia* before and during any intervention, since it might have unexpected effects on the strategy employed [103].

This systematic review summarizes all available knowledge about *Wolbachia* in mosquito species focusing on the Culicidae diverse genera, the methodological approaches employed for *Wolbachia* detection and discuss the different sources of biases that can emerge due to methodological limitations and/or biological symbiotic factors.

### 3.1. Distribution of Wolbachia in Culicidae

The Culicidae family is one of the largest families of insects comprising more than 3574 known species [53]. Only 217 of these were evaluated for the presence of *Wolbachia* so far, which represents 6% of Culicidae species (Figure 1B). Thus, the distribution and frequency of *Wolbachia* in more than 90% of Culicidae species remains unknown, as well as the possible existence of different strains and induced phenotypes in the host mosquito species. As expected, there is a strong correlation between the abundance of mosquito species per genera and the number of species per genera screened for *Wolbachia* (Figure 2). *Anopheles*, *Aedes*, and *Culex*, were the three most studied genera screened for *Wolbachia* comprising 75% of the total. The most studied species was *Ae. albopictus* (44% of articles). Such higher representativeness of these three genera was expected since they are also the most abundant and have enormous epidemiological importance transmitting several pathogens to humans [34,104,105]. However, it is important to note that very few mosquito species from other genera, such as *Haemagogus* and *Sabethes* that also transmit highly pathogenic pathogens, were barely screened for *Wolbachia* [106]. For instance, no species from the *Sabethes* genus was screened for *Wolbachia* so far and only one species from the *Haemagogus* genus was investigated. Other examples of mosquito species with epidemiological importance, such as *Ae. furcifer*, *Ae. taylori*, *Ae. luteocephalus*, and *Ae. simpsoni* that maintain the peridomestic cycle of YFV in different countries on the African continent, have not been screened for *Wolbachia* so far [107].

On the other hand, mosquitoes that belong to the genera *Toxorhynchites*, *Malaya*, and *Topomania* were not investigated at all, maybe because they are not considered of medical importance due to their non-hematophagous feeding habits [106]. However, knowing the *Wolbachia* diversity in these mosquitoes is important to understand the evolutionary history of *Wolbachia* since species from these genera are basal in the phylogeny of culicids. Moreover, in-depth knowledge of the interaction with their hosts may provide new *Wolbachia* strains that can be exploited for biological control. Thus, there are still major gaps in the knowledge about *Wolbachia* diversity in mosquitoes that should be addressed in the coming years.

### 3.2. Detection Methods and What They Can Tell Us

Molecular biology techniques continue to improve as the years pass, many developments have been incorporated to detect different *Wolbachia* molecules and cells in insects. However, each methodology has its strengths and weaknesses that can directly influence the results of *Wolbachia* diagnosis. Some factors must be considered before establishing the method to detect *Wolbachia* in mosquitoes including the sensitivity of the technique; the specificity of the chosen target; the biological characteristics of the bacterium and hosts in different scenarios, such as the possibility of the *Wolbachia* genome being integrated into the mosquito’s genome, tissue tropism of *Wolbachia* infection and variable infection rate (see Section 3.3).

#### 3.2.1. Amplification-Based Strategies

**Figure 4:**
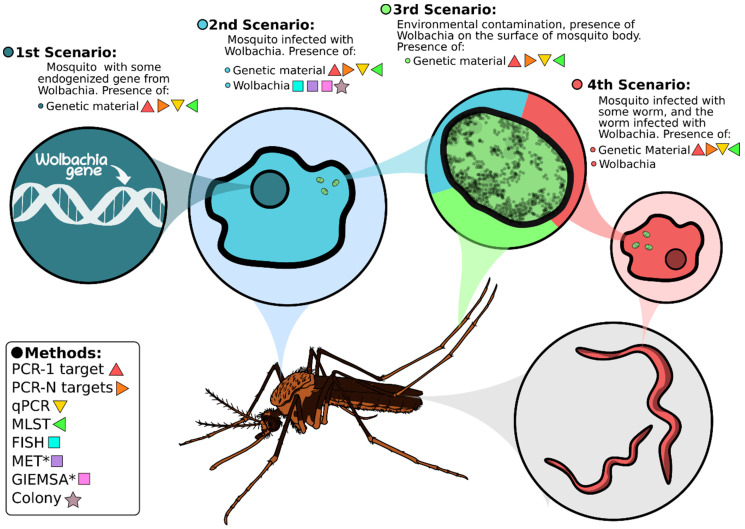
*Wolbachia* detection methods and possible results derived from true infection and/or *Wolbachia* contamination. Triangles and squares represent DNA-based and visualization methods, respectively. Each circle represents one specific scenario and which of the following methods could be used to detect *Wolbachia* molecules. * methods that can suggest the presence of a bacteria establishing a true infection but cannot confirm if this infection is caused by *Wolbachia*.

According to the results found in this review, 33% of the studies performed detection of *Wolbachia* in culicids using conventional PCR with only one *Wolbachia* target gene. Of these, the *wsp* gene that encodes the main protein on the bacterium’s membrane surface, was the main target choice [108]. Although PCR is a sensitive technique that allows the detection of bacterium DNA even at low infection titer, it has some limitations, such as the inability to differentiate if *Wolbachia* DNA was derived from *Wolbachia* cells, a true *Wolbachia* infection, or if *Wolbachia* DNA is integrated into the host genome [109]. Several studies reported cases of lateral gene transfer (LGT) between *Wolbachia* and its hosts, such as the presence of fragments of the *wBruAus* genomic DNA in the X chromosome of the species *Callosobruchus chinensis* and evidence of transfer between the *wMel* strain and *C. chinensis* genome [110,111]. Conventional PCR that amplifies only one target is unable to differentiate a bacterium gene integrated in the mosquito’s genome from an active and true infection by *Wolbachia* (Figure 4).

An alternative to circumvent this problem could be using a large set of *Wolbachia* gene targets (in a multiplex PCR or MLST) since the integration of several bacteria genes in the host’s genome is less likely to occur [110]. Only three of the articles evaluated by this review applied the MLST technique, which consists in amplifying five conserved *Wolbachia* genes widely distributed in the genome, namely: *gatB*, *coxA*, *hcpA*, *fbpA*, and *ftsZ* [112]. Next-generation sequencing (NGS) employing long reads of mosquito and *Wolbachia* DNA may offer additional data that can help to distinguish between a true *Wolbachia* infection and integrated bacterium genomic fragments. Long DNA reads allow the detection of *Wolbachia* DNA integration sites into the mosquito genome or the confirmation of circular *Wolbachia* genomic DNA that further supports a true *Wolbachia* infection hypothesis [46]. However, many genome assembly parameters must be considered when analyzing these genomes, including genome coverage and sequencing depth.

Another alternative is to use qPCR for *Wolbachia* genes, which may indicate a true infection. The bacterium titer variability is most likely explained as a result of a true infection and not a variable amount of *Wolbachia* genes integrated into the genome of different specimens, although *Wolbachia* titer variation may emerge from superficial mosquito contamination. Compared to conventional PCR, the other techniques cited are more laborious and expensive; however, they offer a more precise result for the origin of the detected *Wolbachia* gene.

#### 3.2.2. Cell/Structure Visualization Strategies

Of the 59 articles analyzed, only one used a method for visualizing the bacterium, which was performed using MET. Although molecular techniques to visualize antigens or cell structures, such as FISH and MET are laborious, require expensive equipment and trained personnel, they can discern the first scenario (integration of *Wolbachia* gene into the mosquito genome) from a true *Wolbachia* infection, since the visualization of the bacteria and not just a single structure or gene trace confirm a true infection [46] (Figure 4). An alternative to the high cost required by these cited techniques is to perform a squash of the mosquito’s ovaries followed by staining with May-Grunwald-Giemsa method (GIEMSA) or Gimenez staining to visualize pleomorphic structures suggestive of *Wolbachia* cells, through an optical microscope [61,113,114]. This simpler and cheaper technique was performed by a single study among all investigated in this review. Thus, despite the low amount of resources, there are alternatives for the correct detection of *Wolbachia* in culicids. For example, the use of conventional PCR associated with the visualization of pleomorphic structures in the ovaries of mosquitoes stained by GIEMSA, is able not only to confirm the *Wolbachia* infection but also to detect the specific presence of *Wolbachia* and possibly its lineage or strain.

#### 3.2.3. Laboratory Colony Establishment of Field-Collected Population

The establishment of a colony of field-caught mosquitoes is one of the most complex methodologies to study *Wolbachia* infection. An adequate minimum insectarium structure for colony establishment is required with adjusted temperature, humidity, and specific light/dark cycles adjustments for each species [115,116]. In addition, many species reproduce in particular conditions in the field, such as *An. gambiae* whose males gather in swarms at specific mating sites or species that feed on specific hosts and plants, such as *Uranotaenia macfarlanei* whose preferred source of blood are amphibians [117,118]. Thus, the use of this approach could be required if there was no possibility to confirm an active *Wolbachia* infection by other methodologies, however, given the possibility to use simpler associated molecular methods, the establishment of laboratory colonies becomes a last resource. The establishment of field colonies is so labor-intensive that it was performed by a single study with *Ae. aegypti*, a well-known species regarding basic conditions required to raise and keep a laboratory colony.

#### 3.2.4. Contamination Sources

The contamination of the mosquito samples by *Wolbachia* remains from environmental sources is an important source of bias that should be ruled out before *Wolbachia* infection is determined. Several potential contamination sources have been proposed including the environment, ecto and/or endoparasites [46]. Filarial nematodes of the Onchocercidae family have a mutualistic relationship with *Wolbachia*, whose supergroups C, D, J are present exclusively in these worms [119,120]. Many of these nematodes can be found in mosquitoes since they are involved in their transmission. For instance, *Wuchereria bancrofti* is commonly found in *Cx. quinquefasciatus* populations [121]. Therefore, the detection of *Wolbachia* in mosquitoes could be a result of filarial worm infection instead of a true mosquito infected by *Wolbachia* (Figure 4). The detection of *Wolbachia* strains belonging to supergroups other than A and B (commonly found in mosquitoes) should be taken with caution and further experiments are needed to evaluate the mosquito species infection [20]. Two studies from our systematic review detected *Wolbachia* from supergroups C and D in *Ae. aegypti* and *An. baimai* respectively. In these cases, the two previously mentioned approaches (PCR plus visualization by microscopy methods) are insufficient to differentiate contamination from true *Wolbachia* infection, as these methods do not exclude the possibility of contamination by worms. One alternative experiment to circumvent this scenario would be to perform an additional PCR targeting worm species in the samples considered positive for *Wolbachia*, a negative PCR would add evidence that the *Wolbachia* detection is a result of a true mosquito infection.

Several studies reported *Wolbachia* in *Ae. aegypti*, but the majority used only diagnostic amplicon-based molecular tools [51,54,55,56,57,58] (Supplementary Material). Two studies went further and analyzed the presence of *Wolbachia* through maternal transmission and electron microscopy [45,52], but several criticisms have been made due to the lack of methodological consistency or experimental reproducibility issues [103]. Thongsripong et al., 2018 and Carvajal et al., 2019 detected *Wolbachia* belonging to supergroups C and D in *Ae. aegypti*, but using only amplification-based methods [51,58]. With the lack of further validation with complementary methodologies such results are likely an indication of the presence of contamination by other sources (worms, exuviae, etc.) since these supergroups of *Wolbachia* were not previously found in Diptera [21]. Even if *Wolbachia* is infecting *Ae. aegypti* natural populations, it remains to be assessed if it would induce any phenotype that could interfere with the effectiveness of the *Wolbachia*-based strategies being currently employed. If we consider *Ae. aegypti* bearing worms with *Wolbachia* supergroup C and D, it would mean that *Wolbachia* did not establish an endosymbiotic association with the mosquito and, therefore, no consequences would be expected to control program strategies.

The contamination of *Wolbachia* in mosquito samples can also derive from the external environment in which the mosquitoes insects with plants, water, or niches shared with other infected arthropods [122,123]. *Wolbachia* debris could be acquired by the mosquito feeding and, therefore, two approaches can be taken to differentiate a stable infection from contamination, the first of which is to visualize the bacterium by microscopic methods in mosquito ovaries. The second would be to collect water from breeding sites of the mosquitoes investigated and perform PCR for *Wolbachia* to exclude the possibility of environmental contamination.

Given these examples, it is possible to see that the detection of a true *Wolbachia* infection is a challenging task that can be highly impacted by the choice of the appropriate technique. Thus, the best way to correctly infer *Wolbachia* infection is to choose the best set of complementary techniques that can discern the different possible scenarios regarding the presence and/or infection of *Wolbachia* in mosquitoes (Figure 4).

### 3.3. Wolbachia Detection in Different Mosquito Populations: The Symbiotic Population Dynamics Hypothesis

Several biological phenomena derived from the intricate symbiotic relationship between *Wolbachia* and its hosts must be taken into consideration to interpret the *Wolbachia* detection results using different methodologies. Some species investigated in this review were reported both negative and positive for *Wolbachia* infection. In some cases, contaminated samples, misidentified species or low sensitivity of the technique used to detect *Wolbachia* molecules or cells can lead to divergent results regarding the presence of the bacteria in a given species/population [103]. However, this discrepancy can also be a result of the host–parasite population dynamics itself. *Wolbachia* infection rate is not uniform throughout every population due to a series of environmental and biological factors. Since *Wolbachia* infection is dynamic and mosquito populations can be very large and widely distributed, the infection rate in mosquitoes in each population can be variable, then the sampling of a few individuals from single or few sampling sites might not represent the full dynamics of the *Wolbachia* infection at the population level as a whole. Due to methodological differences employed by studies that investigated populations of the same species, it is not straightforward to find reliable examples of such biases. Only one clear example of the species *Ae. cantans*, from populations from Italy and Russia was detected in this systematic review [85,109]. Both screened *Wolbachia* by PCR targeting the *wsp* gene with the same primer pairs; however, the first one sampled five individuals of the species while the second used approximately 1700 individuals of several subpopulations. As a result, Shaikevich et al., 2019, detected the *wOcan Wolbachia* strain from Russian populations, while Ricci et al., 2002 did not detect any positive samples. Faced with such different sampling efforts, we must ask: are those results derived from a real biological phenomenon of variable infection rate (the symbiotic factor) or the result of the large difference in the analyzed number of specimens/subpopulations? Moreover, due to the lack of complementary molecular techniques to evaluate *Wolbachia* infection into mosquito cells another key question emerges: Is that species really infected by *Wolbachia*? (see Detection methods and what they can tell us section). To avoid sampling bias a minimum number of specimens that is sufficient to represent different populations of the species distributed along the host species niche is necessary to determine whether a species of Culicid does or does not harbor *Wolbachia* naturally and complementary methodologies should be employed to assess the real infectious status of the species.

The minimum number of specimens necessary to accurately assess the infection status of a species is difficult to determine once each host species has different population size and may have different symbiotic relationship with *Wolbachia*. Some mosquito species are present in large population sizes, such as cosmopolitan species *Ae. aegypti* and *Cx. quinquefasciatus*. In this case, a low number of diagnostic specimens will likely not reveal the true *Wolbachia* infection rate [124,125]. While other species are present in limited population size and inhabit very specific niches, such as species from the *Sabethes* and *Haemagogus* genera [126], where a limited number of specimens would be reasonable. On the other hand, the *Wolbachia* symbiotic relationship with the mosquito host can also impact the minimal number of specimens to be investigated since *Wolbachia* strains that can manipulate the host reproductive systems can reach a high infection rate. Hence, few host individuals would be enough to evaluate the infection status of a given host species. While strains that do not induce any phenotype may have a very low infection rate and a much higher number of host specimens would be required to ascertain its infection status. Although there is no general rules to define the minimum number of specimens needed to accurately evaluate the infection status of a giving mosquito species, there are some interesting guidelines on disease ecology that highlight several sources of bias that can be readily transferred to the *Wolbachia*–host relationship dynamic such as Colvin et al., 2015, and Lachish and Murray, 2018 [127,128].

*Ae. aegypti* and *An. gambiae* are two epidemiological relevant species in which the presence of *Wolbachia* has been investigated by several studies [46,103]. So far, six *Ae. aegypti* natural populations from different countries have been *Wolbachia* positive, in addition to a characterization of a new strain (*wAegB* from supergroup B) [45], while several other studies analyzing several populations from 27 different countries, have not identified the presence of *Wolbachia* in this species [129]. Comparing these studies is difficult since none of them used the same methodological approach, with the same *Wolbachia* markers or with the same number of screened mosquitoes. These diverging results can be a result of methodological and/or experimental biases as well as something derived from the population dynamics between symbionts that may be influenced by the environment and interaction with other species. Some studies have demonstrated that high temperature influences the *Wolbachia* titer present in mosquitoes, as observed in *An. stephensi* in the laboratory and in field tests with *Ae. aegypti* [130,131,132]. Such a phenomenon may lead to the elimination of *Wolbachia* infection in a given population. Environmental characteristics are known to influence the establishment of the mosquito microbiota, where populations sampled from different sites show different midgut bacterial composition due to factors such as, breeding water composition, temperature, and anthropogenic activities in these regions [133]. Thus, these factors could also influence the presence of *Wolbachia* in certain species facilitating or preventing the symbiotic establishment between the host and the bacteria. As several species of epidemiological importance are present in regions with different geographical characteristics, the symbiotic population dynamics might explain the divergence for the results regarding the presence of *Wolbachia* in different mosquito populations. A better understanding of how these biological factors influence the presence of *Wolbachia* in mosquitoes is necessary to plan *Wolbachia* infection surveillance in different mosquito populations and guide intervention measures appropriately.

## 4. Materials and Methods

This systematic review follows the criteria established by PRISMA (Preferred Reporting Items for Systematic reviews and Meta-Analyses) and the checklist for reporting systematic reviews and meta-analyses [134].

### 4.1. Search Strategy

A search in the PUBMED platform was performed between March and July 2020 with the following terms: (1) mosquito OR vector OR Culicidae AND *Wolbachia* AND infection, (2) mosquito OR vector OR Culicidae AND *Wolbachia* AND detection, (3) mosquito OR vector OR Culicidae AND *Wolbachia* AND surveillance, (4) mosquito OR vector OR Culicidae AND *Wolbachia* AND distribution. The last search was performed on 2 July 2020.

### 4.2. Eligibility Criteria

The inclusion criteria selected were: articles that detected *Wolbachia* strains in natural populations (directly sampled from the field) of insects of the Culicidae family, regardless of the methodology chosen for detection, study publication year, or region. The exclusion criteria were: (1) review articles, (2) detection of *Wolbachia* in insects other than the Culicidae family, (3) detection of the *Wolbachia* only in cell culture, (4) detection of the bacteria in mosquito colonies, (5) articles that validate a molecular detection technique, (6) articles that detected *Wolbachia* in Culicidae from places where mosquitoes infected with *Wolbachia* were released, (7) articles not published in English language.

These criteria were established to understand the natural distribution and diversity of *Wolbachia pipientis* in culicids of different genera and locations.

### 4.3. Study Selection

**Figure 5:**
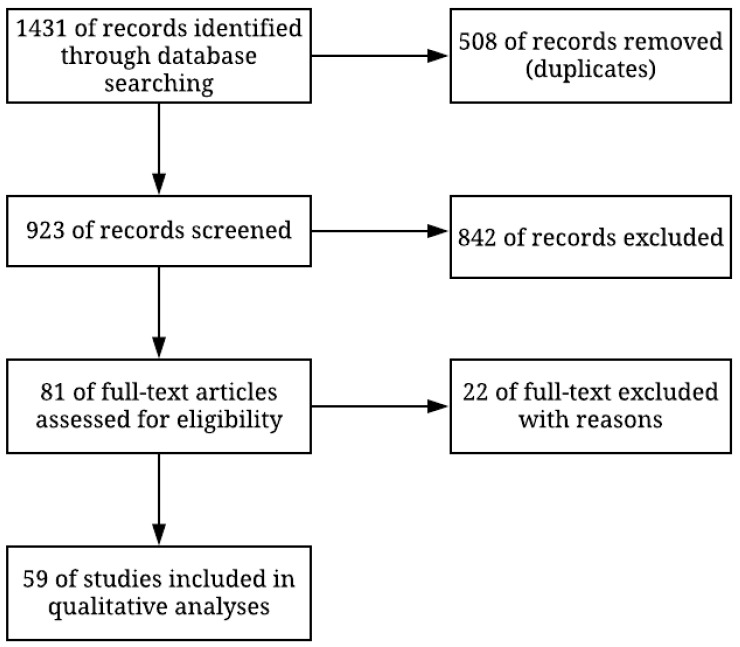
Methodological flowchart showing the number of articles used for this work.

The initial search on National Centers for Biotechnological Information (NCBI) returned 1431 articles. After duplicates and screening using the inclusion and exclusion criteria, 59 articles remained and were used to extract the relevant data (Figure 5).

### 4.4. Data Extraction

These were the data extracted from the selected articles were: (1) title of the article, (2) year of publication, (3) Culicidae species analyzed for *Wolbachia* detection (4) Infection status by *Wolbachia*: positive or negative; in case of positive infection (5) infection rate and (6) *Wolbachia* supergroup or strain, (7) detection method used, and (8) mosquito collection site (Table S1).

The analysis excluded which stages of the mosquito life cycle were used for detection, the different infection rates between males and females (only the general percentage of mosquito infection was retained), and taxonomic classifications lower than species level for culicids.

When the data were unclearly described by the authors, the annotation was performed as without information, for example, the absence of *Wolbachia* supergroups or strains description and the absence of infection rate information. Regarding the sites, only the countries in which the Culicidae collections were provided were recorded, and no other territorial designations, such as cities and/or districts.

Some studies used more than one detection method for *Wolbachia* infection detection, however, the species of culicid was considered positive if *Wolbachia* was detected in at least one of the techniques.

Finally, the nucleic acid sequencing method was not considered as a method to detect *Wolbachia*, but as a method to classify into supergroups or as a way to infer relationships between the bacterium and its hosts.

## 5. Conclusions

*Wolbachia* is an endosymbiotic bacterium with large biotechnological interest for vector/disease control. Despite the great advances and discoveries made on *Wolbachia* mediated host physiological changes, its presence in several species and genera of culicids, is not well described yet. The absence of the correct classification of the bacterium in supergroups as well as the lack of consensus on the establishment of a standard methodology capable of discerning between a true *Wolbachia* infection or other sources of *Wolbachia* molecules, hinder proper comparison between studies and obscure the evolution at species and population level of this bacterium as a whole. Thus, more precise investigations in a wide range of mosquito species must be performed to allow a better understanding of the natural *Wolbachia* infection in Culicids. Such information will be crucial to planning and implementing different strategies that use *Wolbachia* to reduce vector population and/or decrease the public health burden of different mosquito-borne pathogens.

## Figures and Tables

**Table 1:** *Wolbachia* infection rate in different mosquito species investigated.

Species	Minimum (%)	Maximum (%)	Strain	Supergroup	References
*Ad. madagascarica*	-	100	*wMad*	NA	[43]
*Ae. aegypti*	4.3	57.4	*wAegB*, *wAlbB*	A, B, C	[45,51,52,54,55,56,57,58]
*Ae. albopictus*	15	100	*wAlbA*, *wAlbB*, *wPip*	A,B	[49,50,51,54,55,56,57,59,60,61,62,63,64,65,66,67,68,69,70,71,72,73,74,75,76,77]
*Ae. bromeliae*	-	75	NA	NA	[78]
*Ae. cantans*	-	3	*wOcan*	NA	[60]
*Ae. cinereus*	-	37	*wAcin*	NA	[60]
*Ae. metallicus*	-	50	NA	NA	[78]
*An. “GAB−2”*	-	63	NA	B	[79]
*An. “GAB−3”*	-	100	NA	B	[79]
*An. arabiensis*	3.1	7.5	NA	NA	[80,81]
*An. carnevalei*	-	7	NA	A,B	[79]
*An. coluzzii*	3	4	NA	A,B	[79,80]
*An. coustani*	-	6	NA	B	[79]
*An. funestus*	1.21	5	*wAnfuA*, *wAnfuB*	A,B	[79,82]
*An. gambiae*	8	24	*wAnga*	B	[79,80,83]
*An. hancocki*	-	2	NA	B	[79]
*An. implexus*	-	4	NA	B	[79]
*An. jebudensis*	-	50	NA	B	[79]
*An. marshallii*	-	5	NA	B	[79]
*An. moucheti*	-	71	*wAnM*	B	[79,80]
*An. nigeriensis*	-	4	NA	B	[79]
*An. nili*	-	58	NA	B	[79]
*An. paludis*	-	6	NA	B	[79]
*An. species A*	-	91	*wAnsA*	A	[80]
*An. stephensi*	-	60	NA	A,B	[71]
*An. vinckei*	-	10	NA	A,B	[79]
*Ar. kesseli*	8	24	*wKes*	B	[49,59,64]
*Ar. obturban*	-	71	*wPip*	B	[71]
*Ar. subalbatus*	-	100	*wAlbA*, *wSub*, *wRiv*	A	[49,59,61,64,68,84]
*Cq. richiardii*	68	100	*wCrich*	B	[60,62,85]
*Cx. antennatus*	-	3	NA	NA	[43]
*Cx. decens*	-	18	*wDec*	B	[43]
*Cx. dutton*	-	100	NA	NA	[43]
*Cx. gelidus*	-	54	*wGel*, *wCon*	A,B	[49,59,61,64,67,86]
*Cx. hortensis*	-	16.7	NA	NA	[62]
*Cx. modestus*	-	7	NA	NA	[60,85]
*Cx. pipiens*	4.5	100	*wPip*	B	[42,44,60,66,68,84,85,87,88,89,90,91,92,93,94,95,96,97]
*Cx. quinquefasciatus*	30	100	*wPip*	B	[49,51,54,56,59,61,64,66,67,69,72,78,84,88,93,98,99,100,101]
*Cx. theileri*	-	10.4	NA	NA	[70]
*Cx. vishnui*	-	67	*wPip*, *wRiv*, *wCon*	A,B	[49,59,61,64,67,71]
*Fi. circumtestacea*	-	33	*wCir*	NA	[43]
*Ma. africana*	-	27	NA	NA	[78]
*Ma. uniformis*	26	100	*wPip*, *wUnif-Mad*, *wUnifB*, *wRiv*, *wCon*	A,B	[43,49,61,64,67,72,78,84]
*Oc. dorsalis*	-	100	NA	NA	[62]
*Ur.* spp.	-	26	*wUra1*, *wUra2*	A	[43]

NA: not annotated at supergroup or strain level.

## Data Availability

The data presented in this study are available in Supplementary Material.

## Supplementary Materials

The following are available online at https://www.mdpi.com/2076-0817/10/1/39/s1, Table S1: All data used to review the articles, including search terms, articles selected, and positive and negative species.
